# Simultaneous removal of nitrogen and arsenite by heterotrophic nitrification and aerobic denitrification bacterium *Hydrogenophaga* sp. H7

**DOI:** 10.3389/fmicb.2022.1103913

**Published:** 2023-03-03

**Authors:** Xia Fan, Li Nie, Zhengjun Chen, Yongliang Zheng, Gejiao Wang, Kaixiang Shi

**Affiliations:** ^1^State Key Laboratory of Agricultural Microbiology, College of Life Science and Technology, Huazhong Agricultural University, Wuhan, China; ^2^College of Biology and Agricultural Resources, Huanggang Normal University, Huanggang, China

**Keywords:** *Hydrogenophaga*, nitrogen removal, arsenite oxidation, wastewater microbial treatment, cocontamination

## Abstract

**Introduction:**

Nitrogen and arsenic contaminants often coexist in groundwater, and microbes show the potential for simultaneous removal of nitrogen and arsenic. Here, we reported that *Hydrogenophaga* sp. H7 was heterotrophic nitrification and aerobic denitrification (HNAD) and arsenite [As(III)] oxidation bacterium.

**Methods:**

The appearance of nitrogen removal and As(III) oxidation of Hydrogenophaga sp. H7 in liquid culture medium was studied. The effect of carbon source, C/N ratio, temperature, pH values, and shaking speeds were analyzed. The impact of strains H7 treatment with FeCl3 on nitrogen and As(III) in wastewater was assessed. The key pathways that participate in simultaneous nitrogen removal and As(III) oxidation was analyzed by genome and proteomic analysis.

**Results and discussion:**

Strain H7 presented efficient capacities for simultaneous NH_4_^+^-N, NO_3_^−^-N, or NO_2_^−^-N removal with As(III) oxidation during aerobic cultivation. Strikingly, the bacterial ability to remove nitrogen and oxidize As(III) has remained high across a wide range of pH values, and shaking speeds, exceeding that of the most commonly reported HNAD bacteria. Additionally, the previous HNAD strains exhibited a high denitrification efficiency, but a suboptimal concentration of nitrogen remained in the wastewater. Here, strain H7 combined with FeCl3 efficiently removed 96.14% of NH_4_^+^-N, 99.08% of NO_3_^−^-N, and 94.68% of total nitrogen (TN), and it oxidized 100% of As(III), even at a low nitrogen concentration (35 mg/L). The residues in the wastewater still met the V of Surface Water Environmental Quality Standard of China after five continuous wastewater treatment cycles. Furthermore, genome and proteomic analyses led us to propose that the shortcut nitrification-denitrification pathway and As(III) oxidase AioBA are the key pathways that participate in simultaneous nitrogen removal and As(III) oxidation.

## 1. Introduction

Nitrogen (N) and arsenic (As) are well recognized as the most prevalent contaminants in groundwater (Camargo and Alonso, 2006; Upadhyaya et al., 2010; Shankar et al., 2014). Nitrogen pollution of water primarily includes ammonium (NH_4_^+^), nitrate (NO_3_^−^), and nitrite (NO_2_^−^). Of the pollutants, NO_3_^−^, NO_2_^−^, and As are categorized as human carcinogens by the World Health Organization (WHO), and the WHO drinking water limitations for NO_3_^−^-N, NO_2_^−^-N, and As are 11.3 mg/l, 0.91 mg/l, and 10 μg/l, respectively (WHO, 2022). NH_4_^+^ contamination could lead to water eutrophication and respiratory diseases (Wang et al., 2002). The pollution of nitrogen or arsenic is usually caused by the discharge of domestic wastewater, industrial and agricultural wastewater, and the utilization of agricultural fertilizer and arsenic products, leading to the coexistence of them (Fytianos and Christophoridis, 2004; Li et al., 2016, 2021; Shakya and Ghosh, 2021). For example, the Southern Ogallala aquifer was reported to contain 20 mg/l NO_3_^−^-N and 164 mg/l arsenic (Fytianos and Christophoridis, 2004).

Nitrogen pollution depends largely on microbial treatments (Ahn, 2006; Chen et al., 2012). Traditional microbial treatments require different types of bacterial strains for nitrification and denitrification. Of which, the nitrification is catalyzed by autotrophic nitrifying bacteria to oxidize NH_4_^+^-N to NO_3_^−^-N under aerobic conditions, while the denitrification is catalyzed by heterotrophic denitrifying bacteria to reduce NO_3_^−^-N to N_2_ under anaerobic conditions (Ma et al., 2016). The different growth conditions of the above bacteria would result in an additional design, space requirement, increased cost, and program complexity. Fortunately, a new type of bacteria with the ability of heterotrophic nitrification-aerobic denitrification (HNAD) is constantly being reported and studied (Robertson et al., 1985; Guo L. et al., 2013; Ren et al., 2014; Lei et al., 2016). Compared to traditional biological methods, HNAD bacteria could catalyze the nitrification and denitrification processes simultaneously (Seifi and Fazaelipoor, 2012; Zhang et al., 2012). The HNAD bacteria exhibit high denitrification efficiency, and the alkalinity produced in the denitrification process could compensate for the alkalinity consumed in the nitrification process, which could reduce the reagent consumption (Seifi and Fazaelipoor, 2012; Zhang et al., 2012). However, a suboptimal concentration of nitrogen remained in the wastewater after treatment with the HNAD strains (He et al., 2018; Yang et al., 2019; Zhang Y. et al., 2019).

The dominant forms of As in nature are arsenite [As(III)] and arsenate [As(V)] (Rosen et al., 2011; Fisher et al., 2015). As(III) is highly toxic and difficult to remove by adsorption, while As(V) is low in toxicity and can be easily removed (Guan et al., 2009). Therefore, oxidation from As(III) to As(V) is considered a significant strategy in treating As pollution. To date, many As(III) oxidation bacteria have been isolated, such as *Halomonas* sp. HAL1 (Chen et al., 2015), *Agrobacterium tumefaciens* GW4 (Shi et al., 2018) and *Bosea* sp. AS-1 (Lu et al., 2018). Thus, As(III)-oxidizing bacteria are a great means for environmental detoxification and As remediation.

So far, mixed-microbe reactors have been used for the simultaneous removal of nitrate and arsenic (Sun et al., 2010; Peng et al., 2018; Ceballos-Escalera et al., 2021). However, it remains to study how the three types of nitrogen (nitrate, nitrite and ammonium) can be removed at the same time as arsenic. In this study, an HNAD- and As(III)-oxidizing bacterium was identified; *Hydrogenophaga* sp. H7 exhibited simultaneous nitrogen removal and As(III) oxidation capability. The strain H7 was shown to be highly effective in removing nitrogen (nitrate, nitrite and ammonium) and oxidizing As(III) simultaneously in both medium and wastewater. A batch-to-batch system was established to determine the wastewater treatment capacity of nitrogen and As(III) co-contamination by strain H7. Furthermore, The mechanism by which strain H7 participates in nitrogen removal and As(III) oxidation was investigated by genome and proteomic analysis. To the best of our knowledge, *Hydrogenophaga* sp. H7 was the first bacterium to simultaneous remove nitrogen and arsenite, and the removal efficiency remains high across a wide range of conditions. *Hydrogenophaga* sp. H7 provides a novel and highly efficient potential for the bioremediation of nitrogen and arsenic pollution.

## 2. Materials and methods

### 2.1. Strain and media

*Hydrogenophaga* sp. H7 was isolated from a copper/iron mine soil in Daye City, Hubei, China, as previously reported (Fan et al., 2019). The basal medium (BM) was used to enrich strain H7. The nitrification medium (NM) and denitrification media (DM) were used to measure the abilities of strain H7 to remove NH_4_^+^-N or NO_3_^−^-N (DM-1) or NO_2_^−^-N (DM-2), respectively. The simultaneous nitrification and denitrification mixed media (SNDM) were used to measure the abilities of strain H7 to remove NH_4_^+^-N, NO_3_^−^-N and oxidize As(III) (SNDM-1) simultaneously or remove NH_4_^+^-N, NO_2_^−^-N and oxidize As(III) simultaneously (SNDM-2) (see Supplementary material 1 for the detailed composition of these media).

### 2.2. Nitrogen removal and As(III) oxidation assay during cultivation

Strain H7 was cultured in BM medium at 28°C with shaking at 150 rpm until the OD_600_ was approximately 1.0. For the As(III) oxidation assay, 1% (v/v) strain H7 was inoculated in 100 ml of BM medium by adding 30 mg/l As(III). For the nitrogen removal assay, the cells were collected by centrifugation at 8,000 g for 5 min, washed three times with normal saline, and suspended in normal saline to the same OD_600_. The above bacterial suspension was then inoculated (1%, v/v) in 100 ml of NM, DM-1, DM-2, SNDM-1, or SNDM-2 media. The culture samples were cultured at 28°C with shaking at 150 rpm, and samples were taken to measure the OD_600_ and the concentrations of NH_4_^+^-N, NO_3_^−^-N, NO_2_^−^-N, NH_2_OH, total nitrogen (TN), As(III), and As(V) at designated times (see Supplementary material I for the measurement methods) (APHA, 1998; Yang, 1999; Liao et al., 2013; Wang et al., 2013). All of the above experiments were performed in triplicate. The removal or oxidation rate was calculated as (C_0_-C_t_)/t. The removal or oxidation efficiency was calculated as (C_0_-C_t_)/C_0_*100%. C_0_ is the initial concentration, and Ct is the final concentration at time t. All the results are shown in the form of the mean.

### 2.3. Effects of different factors on nitrogen removal and As(III) oxidation

The effects of carbon source, C/N ratios, pH, temperature, and shaking speed on nitrogen removal and As(III) oxidation were investigated by single factor tests. Detalied parameters are as follows: (1) Carbon sources: glucose, 4-hydroxybenzoate (4-HBA), sodium acetate, and sodium citrate. (2) C/N ratios: 3, 5, 8, 10, and 12. (3) pH: 6.0, 7.0, 8.0, 9.0, and 10.0. (4) Temperature: 15°C, 20°C, 28°C, 37°C, and 40°C. (5) Shaking speed: 0 rpm, 50 rpm, 100 rpm, 150 rpm, and 200 rpm (see Supplementary material I for the detailed steps). The culture samples were taken to measure the OD_600_ and the concentrations of NH_4_^+^-N, NO_3_^−^-N, NO_2_^−^-N, TN, As(III), and As(V) at 20 h. All of the above experiments were performed in triplicate.

### 2.4. The application of strain H7 in wastewater

The wastewater was collected from a pig farm in Wuhan City, Hubei, China, after being treated with the Moving Bed Biofilm Reactor reaction (wastewater O1). Glucose (200 mg/l) and As(III) (5 mg/l) were added to the wastewater in this study. For the single cycle test, strain H7 with 10^7^ CFU (colony forming units) was inoculated in 100 ml wastewater to analyze the effect of strain H7 on nitrogen and arsenic removal. The treated wastewater was cultured at 28°C with shaking at 75 rpm for 16 h, and then 108 mg/l FeCl_3_ was added at 16 h. For the batch-to-batch experiment, strain H7 with 10^7^ CFU was inoculated in 100 ml wastewater and incubated at 28°C with shaking at 75 rpm for 18 h. The bacterial cells were collected by centrifugation at 8000 g for 5 min, washed three times with normal saline, and suspended in normal saline. The suspension was then inoculated with 100 ml of fresh wastewater. A total of 108 mg/l FeCl_3_ was added at the end of the cycle. The culture samples were taken to measure the concentrations of NH_4_^+^-N, NO_3_^−^-N, TN, As(III), and As(V) at designated times.

### 2.5. Proteomics preparation and analysis

One experimental group was designed: NH_4_^+^-N versus control (strain H7 cultured in NM medium vs. strain H7 cultured in NM medium, but 45 mg/l urea was the sole nitrogen source). The method of inoculation and culture for strain H7 was consistent with that of the NH_4_^+^-N removal experiment. The cells were collected by centrifugation (8,000 g, 5 min) for 8 h and then freeze-dried, which were detected and analyzed by Wuhan Gene Create Ltd., Wuhan, China (see Supplementary material I for the detailed steps) (Bradford, 1976; Shilov et al., 2007).

### 2.6. Real-time quantitative PCR

qRT-PCR was used to detect the expression of the arsenite oxidase-encoding gene *aioA* in strain H7 (see Supplementary material I for the detailed steps). Gene expression was normalized by 2^−ΔΔCT^ analysis with an iQ5 Real-Time PCR Detection System (Bio-Rad, United States). The primers used in this experiment see Supplementary Table S1.

## 3. Results and discussion

### 3.1. *Hydrogenophaga* sp. H7 removed nitrogen during cultivation

**Figure 1:**
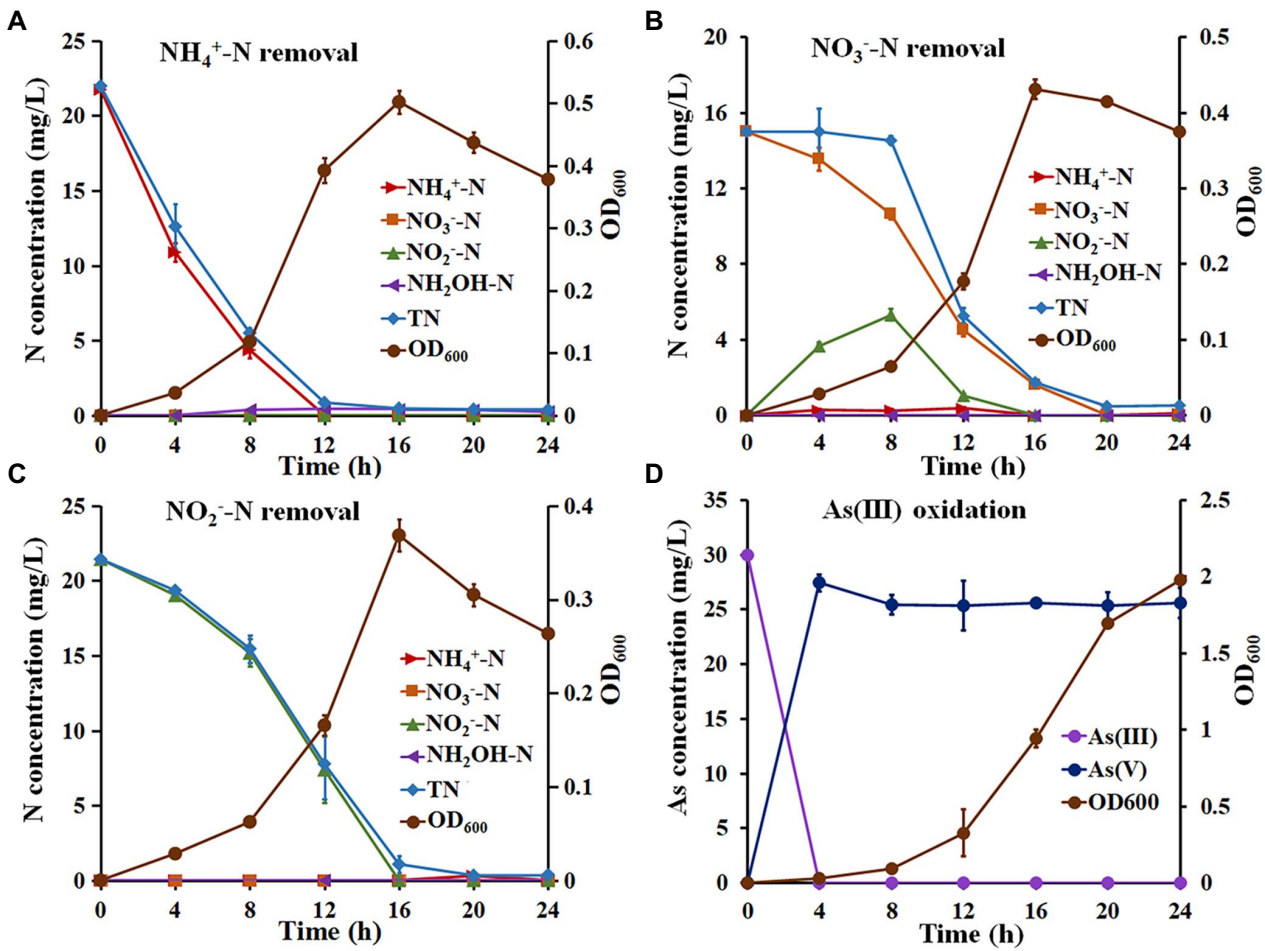
The growth, nitrogen removal, and As(III) oxidation characteristics of strain H7. The growth and nitrogen removal curves of strain H7 using NH_4_^+^-N **(A)**, NO_3_^−^-N **(B)**, and NO_2_^−^-N **(C)** as sole nitrogen sources. **(D)** The As(III) oxidation curves of strain H7. Error bars represent the mean ± standard deviation (*n* = 3).

The NH_4_^+^-N removal capacity of *Hydrogenophaga* sp. H7 is shown in Figure 1A. The OD_600_ reached a maximum value of 0.502 ± 0.019 at 16 h when NH_4_^+^-N was used as a sole nitrogen source. Meanwhile, the removal efficiencies of NH_4_^+^-N and TN reached 99.50% with a maximum removal rate of 2.71 mg/l/h and 97.31% with a maximum removal rate of 2.34 mg/l/h, respectively (Supplementary Table S2). The concentration of hydroxylamine (NH_2_OH), which is the product of nitrification, increased to the maximum value of 1.00 mg/l/h at 12 h (Figure 1A). In addition, strain H7 failed to grow and remove NH_4_^+^-N without the addition of extra organic carbon or carbonate as the sole carbon source (data not shown). These results suggested that strain H7 removed NH_4_^+^-N by heterotrophic nitrification, and its NH_4_^+^-N removal efficiency was higher than that of previous heterotrophic nitrification strains, such as *Bacillus* sp. LY (0.43 mg/l/h) (Zhao et al., 2010), *Pseudomonas* sp. ADN-42 (1.38 mg/l/h) (Jin et al., 2015), *Pannonibacter phragmitetus* B1 (1.16 mg/l/h) (Bai et al., 2019) and *Pseudomonas tolaasii* Y-11 (2.04 mg/l/h) (He et al., 2016). Strain H7 could use NO_3_^−^-N and NO_2_^−^-N as a sole nitrogen source to support its growth and remove them (Figures 1B,C). The OD_600_ of strain H7 reached a maximum value of 0.431 ± 0.012 when NO_3_^−^-N was as a sole nitrogen source (Figure 1B). The removal efficiencies of NO_3_^−^-N and TN by strain H7 were 99.82% with a maximum removal rate of 1.53 mg/l/h, and 97.71% with a maximum removal rate of 2.16 mg/l/h, respectively (Supplementary Table S3). Meanwhile, the concentration of NO_2_^−^-N increased to the maximum value of 5.60 mg/l/h at 12 h with a gradual decrease in NO_3_^−^-N and then decreased to zero at 16 h. These results suggested that strain H7 removed NO_3_^−^-N by aerobic denitrification and that its NO_3_^−^-N removal efficiency was also higher than that of some NO_3_^−^-N removal strains, such as *Pseudomonas putida* P1 (0.68 mg/l/h) (Xiang et al., 2006), *P. phragmitetus* B1 (0.81 mg/l/h) (Bai et al., 2019) and *Rhodococcus* sp. CPZ24 (0.93 mg/l/h) (Chen et al., 2012). The OD_600_ of strain H7 reached a maximum value of 0.369 ± 0.017 when NO_2_^−^-N was used as a sole nitrogen source (Figure 1C). The removal efficiency of NO_2_^−^-N was 100% with a maximum removal rate of 1.95 mg/l/h (Supplementary Table S4), which is higher than that of strains *Pseudomonas* sp. yy7 (0.76 mg/l/h) (Wan et al., 2011), *P. phragmitetus* B1 (0.77 mg/l/h) (Bai et al., 2019) and *Acinetobacter* sp. T1 (1.69 mg/l/h) (Yang et al., 2019). The maximum removal rate of NH_4_^+^-N, NO_3_^−^-N and NO_2_^−^-N of strain H7 and above bacteria was shown in Supplementary Table S5. Meanwhile, the removal efficiency of TN was 97.26% with a maximum removal rate of 1.92 mg/l/h. Taken together, these results suggested that strain H7 removes NO_3_^−^-N and NO_2_^−^-N by denitrification.

### 3.2. *Hydrogenophaga* sp. H7 oxidized As(III) to As(V) during cultivation

The As(III) oxidation ability of strain H7 was then investigated. Strain H7 grew well in BM medium, and its OD_600_ reached 2.0 (Figure 1D). Interestingly, strain H7 could oxidize 30 mg/l As(III) to As(V) within 4 h, while the OD_600_ was less than 0.1 (Figure 1D). The As(III) oxidation rate of strain H7 was 7.5 mg/l/h, which was higher than that of some As(III)-oxidizers, such as *Halomonas* sp. HAL1 (0.31 mg/l/h, OD_600_ > 1.0) (Chen et al., 2015), *A. tumefaciens* GW4 (3.75 mg/l/h, OD_600_ > 0.5) (Wang et al., 2015) and *Bosea* sp. AS-1 (6.25 mg/l/h, OD_600_ > 0.5) (Lu et al., 2018). Our previous study showed that the OD_600_ of strain H7 was also <0.1 when it completely oxidized 30 mg/l As(III) in 1/10 ST medium (Fan et al., 2019). The H7 strain was able to efficiently oxidize As(III) at low biomass, which was beneficial in treating arsenic-contaminated wastewater.

### 3.3. *Hydrogenophaga* sp. H7 simultaneously mediated nitrogen removal and As(III) oxidation during cultivation

**Figure 2:**
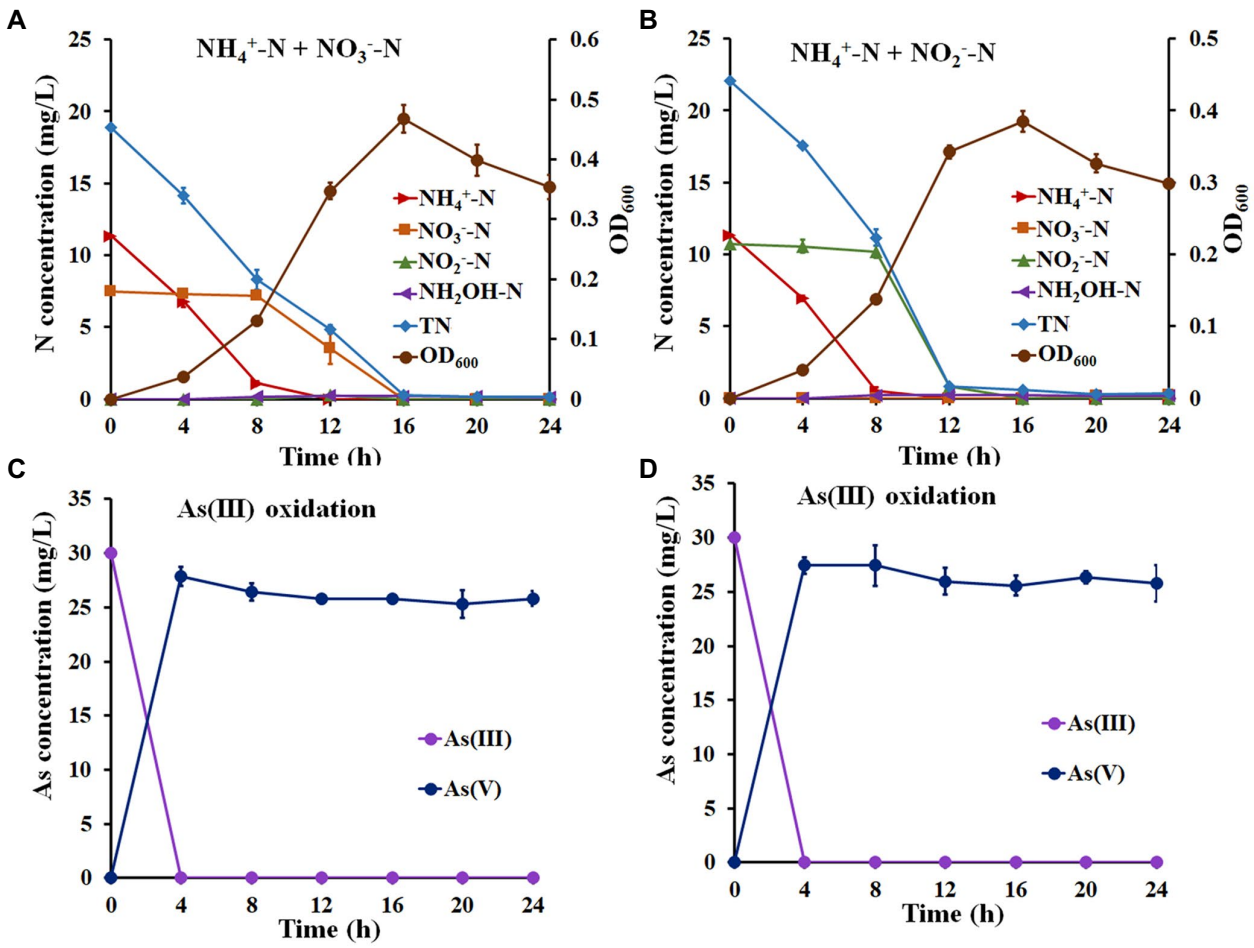
Simultaneous nitrification–denitrification and As(III) oxidation of strain H7. Nitrogen removal **(A)** and As(III) oxidation **(C)** curves of strain H7 in SND-1 medium containing NH_4_^+^-N, NO_3_^−^-N, and As(III). Nitrogen removal **(B)** and As(III) oxidation **(D)** curves of strain H7 in SND-2 medium containing NH_4_^+^-N, NO_2_^−^-N, and As(III). Error bars represent the mean ± standard deviation (*n* = 3).

The above results suggest that strain H7 was a nitrification and denitrification bacterium with As(III) oxidation ability. Therefore, NH_4_^+^-N and NO_3_^−^-N (or NH_4_^+^-N and NO_2_^−^-N) were used as mixed nitrogen sources to investigate the simultaneous nitrogen removal and As(III) oxidation capacity of strain H7. As shown in Figure 2A, the removal efficiency of NH_4_^+^-N was 99.67% with a maximum removal rate of 1.43 mg/l/h, and the efficiency for NO_3_^−^-N was 100% with a maximum removal rate of 0.88 mg/l/h (Supplementary Table S6). In this process, no hydroxylamine or NO_2_^−^-N was detected. Furthermore, the removal efficiency of NH_4_^+^-N or NO_2_^−^-N was 99.53% or 100% with maximum removal rates of 1.62 mg/l/h and 2.33 mg/l/h, respectively (Figure 2B; Supplementary Table S7). Notably, the removal rates of TN in these two experiments reached over 97.90%. Moreover, strain H7 could completely oxidize As(III) to As(V) in 4 h under the above conditions (Figures 2C,D). Taken together, these findings suggest that strain H7 was an HNAD bacterium capable of removing nitrogen and oxidizing As(III) simultaneously.

For the nitrogen removal assays, most of the NH_4_^+^-N-type nitrogen was removed in the first 8 h, while NO_3_^−^-N and NO_2_^−^-N were removed from 8 h to 16 h (Figures 2A,B). These results reveal that strain H7 may be removing NH_4_^+^-N in a mixed nitrogen source before NO_3_^−^-N and NO_2_^−^-N, which might be due to the higher enzyme activity of NH_4_^+^-N oxidization than that of NO_3_^−^-N reduction and NO_2_^−^-N reduction (Shi et al., 2013). In addition, strain H7 removed 11.32 mg/l NH_4_^+^-N from the mixed nitrogen source within 12 h, while it took only 12 h to remove 22.63 mg/l NH_4_^+^-N from the sole nitrogen source (Figures 1A, 2A,B), indicating that additional NO_3_^−^-N or NO_2_^−^-N may inhibit the removal of NH_4_^+^-N by strain H7. These results were consistent with the conclusions from strains *P. putida* ZN1 (Zhang N. et al., 2019) and *Paracoccus versutus* LYM (Shi et al., 2013).

It is well known that NO_2_^−^-N is toxic to humans (Bednarek et al., 2014). Several denitrified bacteria, such as *P. putida* ZN1 (71.57%) (Zhang N. et al., 2019), *P. phragmitetus* B1 (98.73%) (Bai et al., 2019), and *Acinetobacter* sp. T1 (57%) (Yang et al., 2019) could not remove NO_2_^−^-N completely. In addition, some denitrified bacteria, such as *Enterobacter* sp. Z1 and *Klebsiella* sp. Z2, had difficulty completely removing NO_2_^−^-N generated by the reduction of NO_3_^−^-N during denitrification (Zhang Y. et al., 2019). However, strain H7 could completely remove NO_2_^−^-N in either the sole nitrogen source or mixed nitrogen sources. Therefore, strain H7 has certain advantages in the removal of NO_2_^−^-N.

### 3.4. Effects of different factors on nitrogen removal and As(III) oxidation

#### 3.4.1. Carbon source

**Figure 3:**
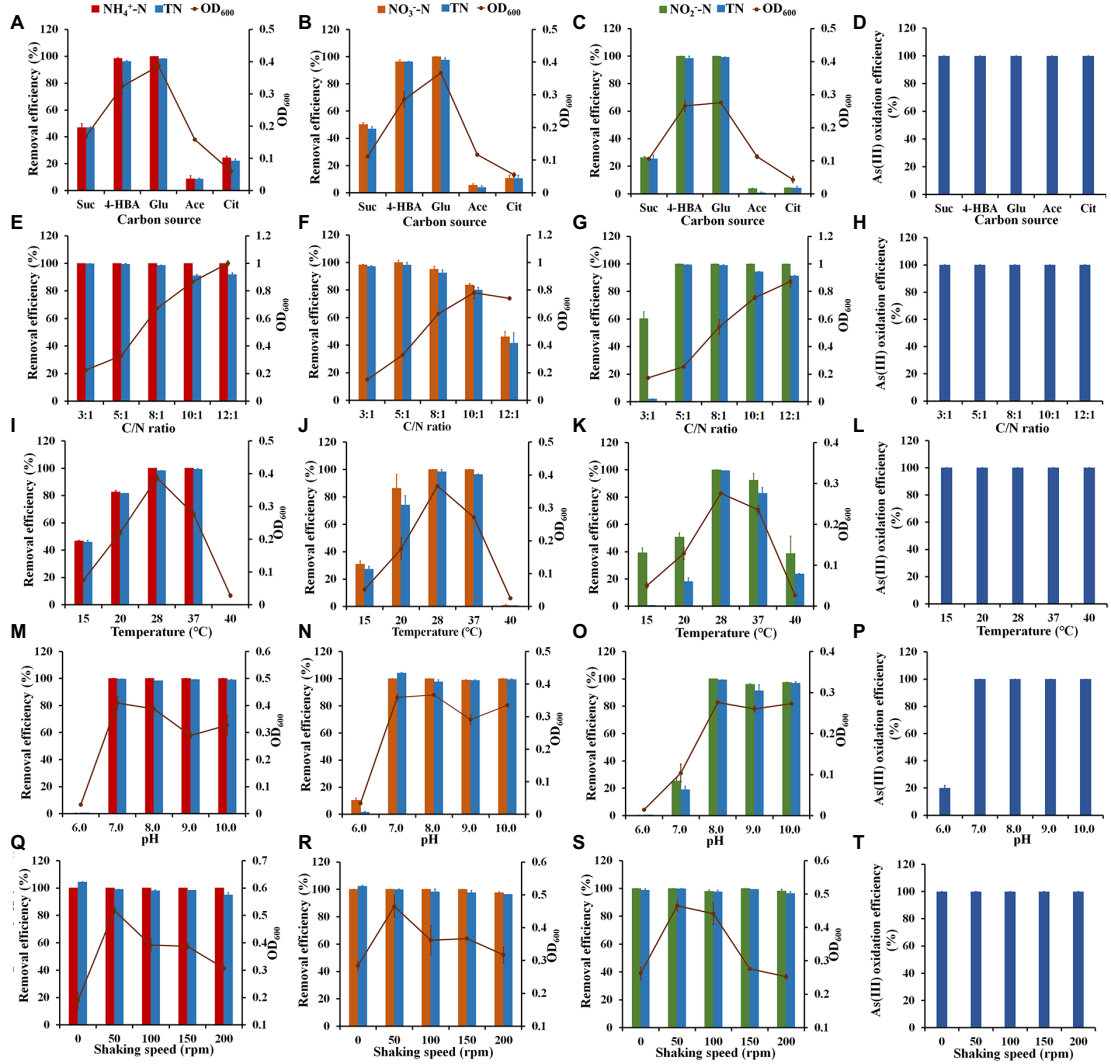
The effects of different factors on nitrification, denitrification, and As(III) oxidation of strain H7. **(A–D)** The effect of the carbon source on nitrogen removal and As(III) oxidation. **(E–H)** The effect of C/N on nitrogen removal and As(III) oxidation. **(I–L)** The effect of temperature on nitrogen removal and As(III) oxidation. **(M–P)** The effect of pH on nitrogen removal and As(III) oxidation. **(Q–T)** The effect of shaking speed on nitrogen removal and As(III) oxidation. A, D, E, H, I, L, M, P, Q, and T used NH_4_^+^-N as the sole nitrogen source. B, F, J, N, and R used NO_3_^−^-N as a sole nitrogen source. C, G, K, O, and S used NO_2_^−^-N as the sole nitrogen source. Suc, sodium succinate; 4-HBA, 4-hydroxybenzoate; Glu, glucose; Ace, sodium acetate; Cit, sodium citrate. Error bars represent the mean ± standard deviation (*n* = 3). Some lines had no error line as a result of the value was 100%.

Carbon sources were used as energy sources and electron donors to affect the growth of heterotrophic bacteria, the denitrification process and bacterial As(III) oxidation (Nandre et al., 2017; Lu et al., 2018). Strain H7 could use sodium succinate, 4-HBA, glucose, sodium acetate, or sodium citrate as the sole carbon source for growth (Figures 3A–C). Strain H7 grew better in the glucose or 4-HBA as a sole carbon source, and the removal efficiencies for NH_4_^+^-N, NO_3_^−^-N, NO_2_^−^-N and TN were all greater than 96.0% (Figures 3A–C). When sodium acetate and sodium citrate were used as the sole carbon source, the removal efficiencies of the above nitrogen sources were less than 25% (Figures 3A–C). These results reveal that strain H7 had a good denitrification capability under glucose or 4-HBA as a sole carbon source. In addition, strain H7 could completely oxidize As(III) at 20 h in the condition of the above five carbon sources (Figure 3D). Consequently, given the growth, nitrogen removal, and As(III) oxidation of strain H7, glucose was chosen as the sole source of carbon for subsequent experiments.

#### 3.4.2. C/N ratio

The effects of the different C/N ratios on nitrogen removal and As(III) oxidation of strain H7 are shown in Figures 3E–H. The removal efficiency of NH_4_^+^-N by strain H7 exceeded 99.5% under different C/N ratio conditions, while the corresponding TN removal exceeded 97.07% (3:1, 5:1 and 8:1) and 91.05% (10:1 and 12:1) (Figure 3E). Additionally, the maximal NO_3_^−^-N and TN removal occurred at a C/N ratio of 5:1 (Figure 3F), but a further increase in the C/N ratio led to decreases in nitrogen removal, which was consistent with *Pseudomonas putida* AD-21 (Kim et al., 2008) but different from *Pseudomonas taiwanensis* strain J, in which the removal of NO_3_^−^-N and TN were comparatively constant (He et al., 2018). Moreover, NO_2_^−^-N could be completely removed under all the above conditions, and the removal efficiencies of TN exceeded 97.26% (5:1 and 8:1) and 91.36% (10:1 and 12:1) (Figure 3G). In particular, when the C/N ratio was 3:1, only 62.3% of NO_2_^−^-N or 2.01% of TN was removed (Figure 3G). This was mainly due to the removal of NO_2_^−^-N converted to NO_3_^−^-N. The As(III) could be oxidized by strain H7 at 20 h in the conditions of all different C/N ratios (Figure 3H). In summary, the best removal and oxidation effect of the above nitrogen sources by strain H7 was a C/N ratio of 5:1.

#### 3.4.3. Temperature

Temperature has an important effect on bacterial growth and metabolism. As shown in Figures 3I,J, the maximum removal of NH_4_^+^-N and NO_3_^−^-N reached 99.5% and the removal of the corresponding TN also exceeded 97.16% between 28°C and 37°C (Figures 3I,J). As shown in Figure 3K, the maximum removal efficiency of NO_2_^−^-N reached 100% at 28°C with a TN removal of 97.26%. The removal efficiencies of NO_2_^−^-N and TN decreased to 92.8 and 82.64%, respectively, at 37°C. Additionally, strain H7 removed 38.61% of NO_2_^−^-N at 40°C, but the high temperature had no effect on the removal of NH_4_^+^-N and NO_3_^−^-N. Moreover, the oxidation of As(III) was finished between 15°C and 40°C even though strain H7 grew weakly at 40°C (OD_600_ = 0.028 ± 0.005) (Figure 3L), suggesting that strain H7 could oxidize As(III) under the low biomass conditions mentioned above (Figure 1D). In summary, the optimum culture temperature of strain H7 for nitrogen removal was 28°C, which falls within the optimal temperature range for most HNAD bacteria (25–37°C) (Rajta et al., 2020).

#### 3.4.4. Initial pH

Bacterial growth and metabolism are closely related to pH. Figures 3M–O shows that the pH had the same influence trend on the growth and nitrogen removal of strain H7. As the pH increased from 7.0 to 10.0, the removal efficiencies for NH_4_^+^-N and NO_3_^−^-N all exceeded 99.5% and that for TN all exceeded 97.0% (Figures 3M,N). The removal efficiencies of NO_2_^−^-N and TN exceeded 96.81% except at pH 6.0 (7.0%) (Figure 3O). Additionally, the efficiencies of As(III) oxidation reached 100% except at pH 6.0 (20.12%) (Figure 3P). These results showed that strain H7 was more suitable for growth, nitrogen removal, and As(III) oxidation in an alkaline environment. At present, the most reported HNAD bacteria that remove nitrogen are in neutral or slightly alkaline environments (Guo Y. et al., 2013; Chen et al., 2014; Yang et al., 2019). However, the removal efficiencies of the above nitrogen sources were still over 98.3% even though the pH was 10.0. This allowed strain H7 to be applied in alkaline environment to remediate nitrogen pollution.

#### 3.4.5. Shaking speed

Shaking speed is one of the key factors in aerobic denitrification. As shown in Figures 3Q–S, a total of 99.5% of NH_4_^+^-N, 97.44% of NO_3_^−^-N and 98.1% of NO_2_^−^-N, respectively, could be removed at shaking speeds from 0 to 200 rpm, and the removal of corresponding TN reached 94.0, 96.35 and 96.3%, respectively. The efficiencies of As(III) oxidation all reached 100% under different conditions (Figure 3T). These results showed that the shaking speed had little effect on strain H7 to remove nitrogen and oxidize As(III). The higher shaking speed during the denitrification process reflects a higher concentration of dissolved oxygen is a critical parameter for effective nitrogen removal (Rajta et al., 2020).

Currently, the removal efficiency of nitrogen for most the reported HNAD bacteria is negatively impacted by the low shaking speed, and too low shaking speed can inhibit the nitrogen removal efficiency from bacteria (He et al., 2018; Chen et al., 2019; Zhang N. et al., 2019; Huang et al., 2022). Interestingly, the different shaking speed conditions (0–200 rpm) had no effect on the nitrogen removal efficiencies of strain H7. Dissolved oxygen is normally low in groundwater, and oxygen would be added to contaminated groundwater to increase nitrogen removal efficiency. Thus, strain H7 has a benefit in groundwater with low dissolved oxygen to remove nitrogen. Moreover, the most bacteria that remove nitrogen occur in neutral or mildly alkaline environments (pH 7–8), but the strain H7 nitrogen removal efficiency has been exceeded by 98% even at a pH of 10.0.

### 3.5. Simultaneous nitrogen removal and As(III) oxidation in wastewater were achieved with strain H7

**Figure 4:**
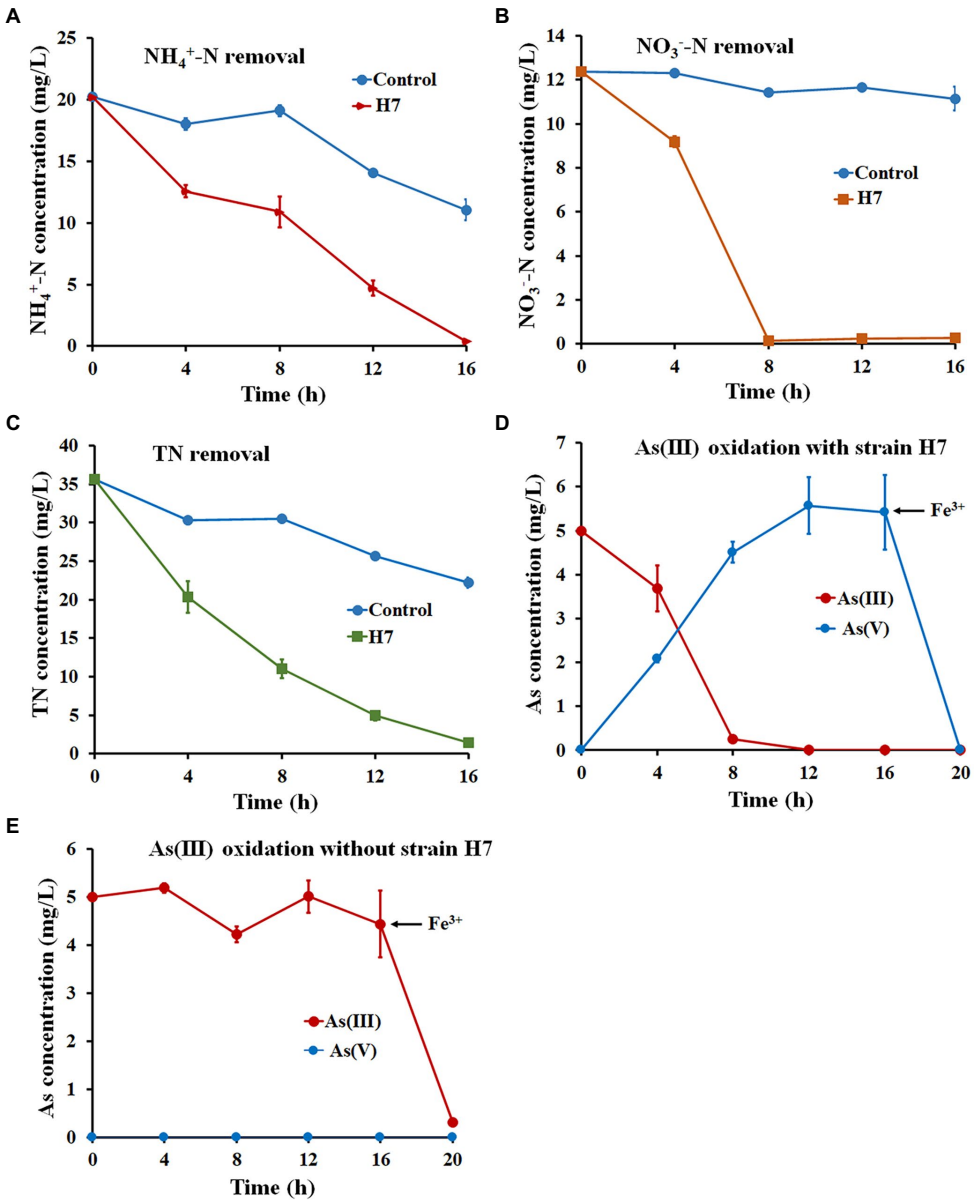
Nitrogen and As(III) removal by strain H7 combined with Fe^3+^ in wastewater. The removal curve of NH_4_^+^-N **(A)**, NO_3_^−^-N **(B)**, and TN **(C)** by strain H7. **(D)** The oxidation and removal curves of As(III) with the addition of strain H7. **(E)** The oxidation and removal curves of As(III) without adding strain H7. Error bars represent the mean ± standard deviation (n = 3).

Wastewater O1 was chosen to analyze the ability of strain H7 to remove nitrogen and oxidize As(III) simultaneously in industrial wastewater. The main characteristics of wastewater O1 are listed as follows: pH 7.82, 20.21 ± 0.21 mg/l NH_4_^+^-N, 12.38 ± 0.33 mg/l NO_3_^−^-N, 35.6 ± 0.55 mg/l TN, and 0 mg/l NO_2_^−^-N. As shown in Figure 4, 98.11% of NH_4_^+^-N, 98.12% of NO_3_^−^-N, and 95.87% of TN were removed, and As(III) was completely oxidized to As(V) with the addition of strain H7 after 16 h (Supplementary Table S8). Fe^3+^ was added and led to the full removal of As(V) within 2 h (Figure 4D). The concentrations of NH_4_^+^-N, NO_3_^−^-N, TN, and As remained at 0.38 mg/l, 0.26 mg/l, 1.46 mg/l, and 0 mg/l, respectively. However, only 45.31% of NH_4_^+^-N, 10.06% of NO_3_^−^-N, and 31.92% of TN were removed, and As(III) was not oxidized to As(V) in the wastewater O1 control without adding strain H7 (Figure 4E), indicating the chemical oxidation will not be occur by dissolved oxygen. Adding Fe^3+^ removed 93.6% of As(III) in the wastewater O1 control, but the residual As(III) concentration (0.32 mg/l) did not reach the integrated wastewater discharge standard (GB3838-2002). These results indicate that strain H7 has great application prospects for remediating nitrogen and arsenic in co-contaminated wastewater.

The ability to remove nitrogen is one of the keys to remediating multiple nitrogen and arsenic contaminations. To date, several HNAD bacteria have been applied to real wastewater. For example, the removal efficiencies of NH_4_^+^-N, NO_3_^−^-N, and TN by *Pseudomonas mendocina* TJPU04 in industrial wastewater were 91% (127 mg/l), 52% (64 mg/l), and 75% (190 mg/l), respectively (He et al., 2018) and *Klebsiella* sp. Z2 removed 95.14% (980 mg/l) of NH_4_^+^-N and 93.37% (1,000 mg/l) of TN (Zhang Y. et al., 2019). Relatively low concentrations of nitrogen remained even in these strains with a high denitrification efficiency, thus failing to meet the Surface Water Environmental Quality Standard of China (GB3838-2002). In this study, the wastewater O1 collected from a pig farm that had been treated by the MBBR reaction belonged to this lower nitrogen concentration type. Thus, strain H7 has great application potential to remediate wastewater with low nitrogen concentrations.

### 3.6. Strain H7 exhibited a batch cycle and stable capacities in nitrogen removal and As(III) oxidation in wastewater

**Figure 5:**
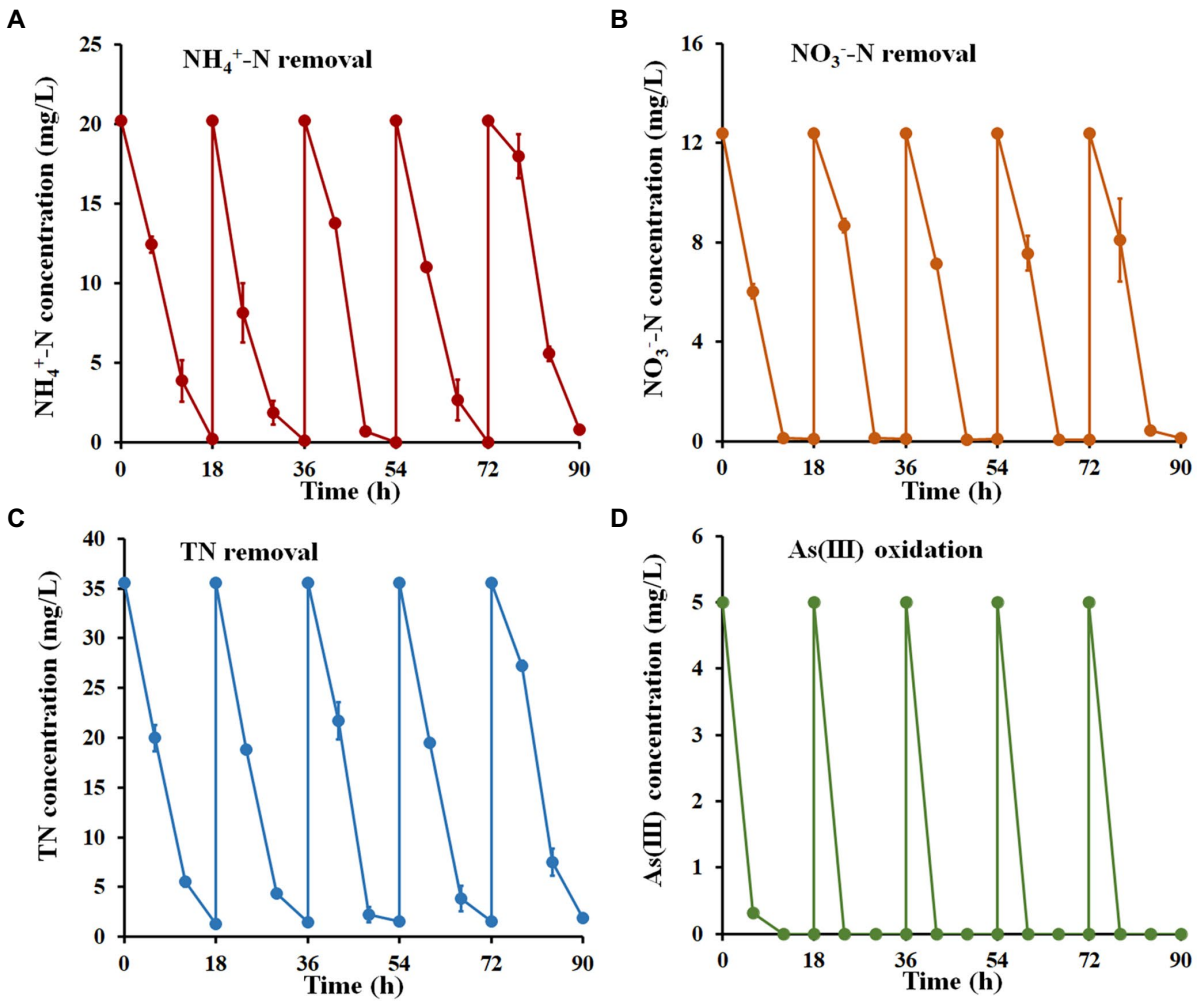
Nitrogen removal and As(III) oxidation by strain H7 in wastewater from batch-to-batch cycles. NH_4_^+^-N **(A)**, NO_3_^−^-N **(B)**, TN **(C)** removal, and As(III) oxidation **(D)** curves of strain H7. Error bars represent the mean ± standard deviation (*n* = 3).

To further identify the ability of strain H7 to remediate nitrogen and As(III) co-contamination, five cycles of a batch-to-batch system were performed. As shown in Figure 5, the removal efficiency for NH_4_^+^-N, NO_3_^−^-N, and TN reached over 96.14, 99.08, and 94.68%, respectively, within 18 h in each cycle. As(III) was completely oxidized to As(V) in each cycle and was removed by the subsequent addition of Fe^3+^. After five cycles, the concentrations of NH_4_^+^-N, NO_3_^−^-N, TN, and As(III) remaining in wastewater O1 were 0.78 mg/l, 0.11 mg/l, 1.89 mg/l, and 0 mg/l, respectively, which still met the V level of Surface Water Environmental Quality Standard of China (GB3838-2002). These results suggested that strain H7 showed good continuity and effectiveness in the bioremediation of nitrogen and arsenic co-contaminated wastewater.

### 3.7. Genome and proteomics analysis of nitrogen removal and As(III) oxidation pathways in strain H7

**Figure 6:**
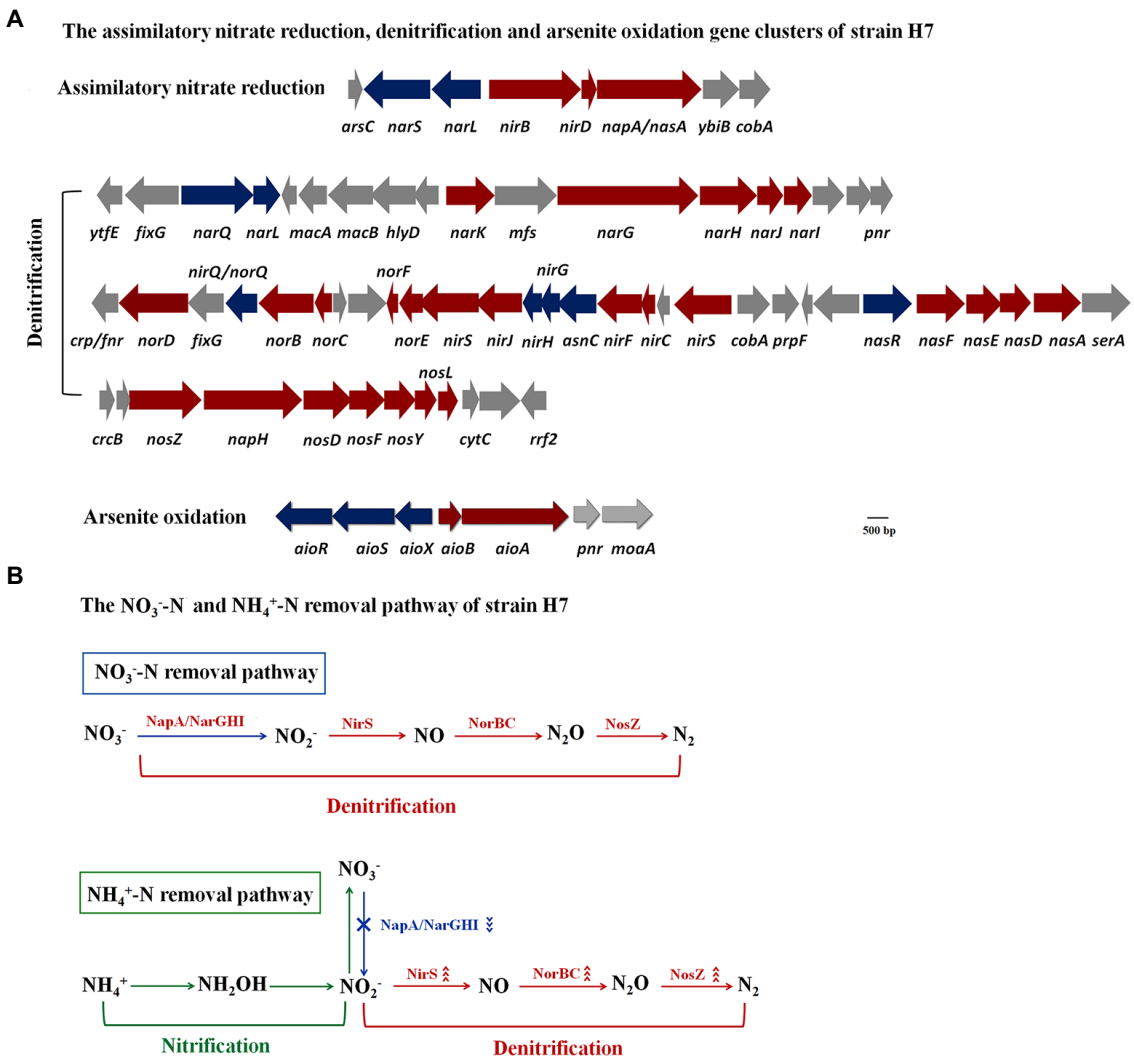
The assimilatory nitrate reduction, denitrification, and arsenite oxidation gene clusters **(A)** and the NO_3_^−^-N and NH_4_^+^-N removal pathways of strain H7 **(B)**.

The genome of strain H7 had been reported in our previous study (Fan et al., 2019). Analyzing the genomic data combined with the Kyoto Encyclopedia of Genes and Genomes, it contained the nitrate reductase gene *nasA* and nitrite reductase gene *nirBD,* which participated in the assimilation of nitrate reduction (Figure 6A). This was consistent with the phenotype that strain H7 could use NO_3_^−^-N or NO_2_^−^-N as a sole nitrogen source to grow. In addition, a complete denitrification pathway was found, including the aerobic *napA* and anaerobic nitrate reductase gene *narGHI*, nitrite reductase gene *nirS*, nitric oxide reductase gene *norBC,* and nitrous oxide reductase gene *nosZ* (Figure 6A), which was consistent with the phenotype that strain H7 could remove NO_3_^−^-N and NO_2_^−^-N. Therefore, the NO_3_^−^-N removal pathway was speculated, as shown in Figure 6B. In addition, an As(III) oxidation island was also found in the strain H7 genome (Figure 6A), and *aioA* was upregulated 2.2-fold by qRT-PCR (Supplementary Figure S2), which was consistent with the phenotype that strain H7 was able to oxidize As(III). The detailed gene information is shown in Supplementary Table S9.

To explore the NH_4_^+^-N removal pathway of strain H7, iTRAQ was performed. NH_4_^+^-N vs. control was designed. A total of 568 proteins showed differential expression; 66 proteins were upregulated, and 182 proteins were downregulated. Detailed information regarding the differentially expressed proteins related to nitrogen metabolism is shown in Supplementary Table S10.

Analysis of the proteins related to nitrogen removal showed that NirS, NorB, NorC, and NosZ were upregulated 3.1-, 5.3-, 2.5-, and 1.7-fold, respectively. However, the NO_3_^−^-N reductases NapA, NarG, NarH, and NarI were downregulated 1.9-, 2.0-, 1.5-, and 1.7-fold, respectively. Usually, the NH_4_^+^-N removal pathway by HNAD bacteria is as follows: (1) NH_4_^+^-N was oxidized to NH_2_OH by ammonia monooxygenase AMO; (2) NH_2_OH was oxidized to NO_2_^−^-N by hydroxylamine oxidase HAO; (3) NO_2_^−^-N is NO_3_^−^-N by NO_2_^−^-N oxidase; (4) NO_3_^−^-N was reduced to NO_2_^−^-N by NapA or NarGHI; (5) NO_2_^−^-N was reduced to NO by NirS; (6) NO was reduced to N_2_O by NorBC; (7) N_2_O was reduced to N_2_ by NosZ (Yang et al., 2019). Nitrification occurred from the first step to the third step, and denitrification occurred from the fourth step to the seventh step. NH_4_^+^-N removal could occur *via* the nitrate pathway (NH_4_^+^ → NO_3_^−^ → NO_2_^−^ → NO→N_2_O → N_2_) or nitrite pathway (NH_4_^+^ → NO_2_^−^ → NO→N_2_O → N_2_) (Yang et al., 2019). In the NH_4_^+^-N removal process of strain H7, no NO_2_^−^-N or NO_3_^−^-N accumulated. The nitrite-reducing NirS, nitric oxide reductase NorBC, and nitrous oxide reductase NosZ were all upregulated, while the nitrate reductases NapA and NarGHI were downregulated. Thus, the NH_4_^+^-N removal pathway of strain H7 occurred *via* the nitrite pathway, which is called shortcut nitrification–denitrification (Figure 6B). Compared with the nitrate pathway, it was reported that shortcut nitrification–denitrification can reduce over 40% of the carbon source addition and 25% of the oxygen supply and greatly improve the denitrification rate (Mavinic and Turk, 1987; Kornaros et al., 2010).

## 4. Conclusion

In this study, *Hydrogenophaga* sp. H7 can simultaneously remove nitrogen and oxidize As(III) in medium and wastewater. Strain H7 exhibited a stable role in simultaneous nitrogen removal and As(III) oxidation over a wide range of temperatures, pH values, and shaking speeds, which is superior to that of the most commonly reported HNAD bacteria. More importantly, combined with Fe^3+^ in wastewater, strain H7 simultaneously removed 94.68% of the total nitrogen and oxidized 100% of As(III) at low nitrogen concentrations. The residual amounts of total nitrogen and arsenic met the V level of Surface Water Environmental Quality Standard of China. Proteomic and genomic approaches reveal that the shortcut nitrification–denitrification pathway and the As(III) oxidase AioBA catalyze simultaneous nitrogen removal and As(III) oxidation. *Hydrogenophaga* sp. H7 provides a novel and highly efficient potential for the bioremediation of nitrogen and arsenic pollution.

## Data availability statement

The mass spectrometry proteomics data of this study have been deposited to the ProteomeXchange Consortium (http://proteomecentral.proteomexchange.org) via the iProX partner repository, accession number PXD038710.

## Author contributions

XF: conceptualization, investigation, writing – original draft, and validation. LN: investigation. ZC: resources. YZ: conceptualization. GW and KS: conceptualization and writing – review and editing. All authors contributed to the article and approved the submitted version.

## Funding

This study was supported by the National Natural Science Foundation of China (no: 32100102 and no: 31870086) and the Open Funds of the State Key Laboratory of Agricultural Microbiology (no: AMLKF202007).

## Conflict of interest

The authors declare that the research was conducted in the absence of any commercial or financial relationships that could be construed as a potential conflict of interest.

## Publisher’s note

All claims expressed in this article are solely those of the authors and do not necessarily represent those of their affiliated organizations, or those of the publisher, the editors and the reviewers. Any product that may be evaluated in this article, or claim that may be made by its manufacturer, is not guaranteed or endorsed by the publisher.
