# Supplementary material for: Simultaneous removal of nitrogen and arsenite by heterotrophic nitrification and aerobic denitrification bacterium Hydrogenophaga sp. H7
Source: Front Microbiol. 2023 Mar 3;13:1103913. doi: 10.3389/fmicb.2022.1103913 (PMC10020585; doi:10.3389/fmicb.2022.1103913)
Supplement: Supplementary file 1 [file Data_Sheet_1.doc]

**Simultaneous removal of nitrogen and arsenite by heterotrophic nitrification and aerobic denitrification bacterium *Hydrogenophaga* sp. H7**

Xia Fan^1,2^, Li Nie^1^, Zhengjun Chen^1^, Yongliang Zheng^2^, Gejiao Wang^1^* and Kaixiang Shi^1^*

^1^*State Key Laboratory of Agricultural Microbiology, College of Life Science and Technology, Huazhong Agricultural University, Wuhan 430070, P.R. China*

^2^*College of Biology and Agricultural Resources, Huanggang Normal University, Huanggang 438000,* *P.R. China*

**Materials and methods**

**Medium**

The basal medium (BM), used to enrich strain H7, contained 0.4 g of (NH_4_)_2_SO_4_, 4.0 g of glucose, 10.6 g of Na_2_HPO_4_∙12H_2_O, 0.8 g of KH_2_PO_4_, 0.1 g of MgSO_4_·7H_2_O and 2 mL of trace elements per liter. The nitrification medium (NM) contained 0.1 g of (NH_4_)_2_SO_4_, 0.5 g of glucose, 10.6 g of Na_2_HPO_4_∙12H_2_O, 0.8 g of KH_2_PO_4_, 0.1 g of MgSO_4_·7H_2_O and 2 mL trace elements per liter. The denitrification medium (DM) contained 0.1 g of KNO_3_ (DM-1) or 0.1 g of NaNO_2_ (DM-2), 0.5 g of glucose, 10.6 g of Na_2_HPO_4_∙12H_2_O, 0.8 g of KH_2_PO_4_, 0.1 g of MgSO_4_·7H_2_O and 2 mL trace elements.

The simultaneous nitrification and denitrification mixed media (SNDM-1 or SNDM-2) contained the following components (per liter): 0.05 g of (NH_4_)_2_SO_4_ and 0.05 g of KNO_3_ (DM-1) or 0.05 g of (NH_4_)_2_SO_4_ and 0.05 g of NaNO_2_ (DM-2), 0.5 g of glucose, 10.6 g of Na_2_HPO_4_∙12H_2_O, 0.8 g of KH_2_PO_4_, 0.1 g of MgSO_4_·7H_2_O, 2 mL trace elements and 30 mg/L As(III) (NaAsO_2_) per liter. Trace elements contained 57.1 g of EDTA∙2Na∙2H_2_O, 3.92 g of ZnSO_4_∙7H_2_O, 7.28 g of CaCl_2_∙2H_2_O, 5.06 g of MnCl_2_∙4H_2_O, 5 g of FeSO_4_∙7H_2_O, 1.57 g of CuSO_4_∙5H_2_O, 1.61 g of CoCl_2_∙6H_2_O and 1.1 g of (NH_4_)_6_Mo_7_O_24_∙4H_2_O per liter. (NH_4_)_2_SO_4_, KNO_3_, NaNO_2_, As(III), and glucose were filtered through a 0.22 μm filter, and other components of the media were autoclaved at 121 °C for 20 min.

**The measurement method of nitrogen and arsenic**

The OD_600_ was measured using a spectrophotometer (UV1900; AOE, China). The concentrations of NH_4_^+^-N and NO_2_^-^-N were determined by Nessler spectrophotometry (APHA, 1998) and the N-(1-naphthalene)-diaminoethane photometry method (Wang et al., 2013), respectively. The concentrations of TN and NO_3_^-^-N were determined by the alkaline potassium persulfate digestion-UV spectrophotometric method (He et al., 2016). The concentrations of NH_2_OH were determined by spectrophotometric method in the presence of Fe(III)-phenanthroline complex (Yang, 1999). The amounts of As(III) and As(V) were detected using high-performance liquid chromatography combined with hydride-generation atomic fluorescence spectroscopy (HPLC-HG-AFS; Beijing Titan Instruments) (Liao et al., 2013).

**Effects of different factors on nitrogen removal and As(III) oxidation**

The same inoculation method that was used for the nitrogen removal performance of strain H7 was also used for the effects of different factors on its removal of nitrogen and oxidation of As(III). For carbon source tests, glucose in NM, DM-1, or DM-2 media was replaced by sodium succinate, 4-hydroxybenzoate (4-HBA), sodium acetate, and sodium citrate, respectively. For C/N ratio tests, glucose was used as the sole carbon source with C/N ratios of 3, 5, 8, 10, and 12 and a fixed concentration (100 mg/L) of (NH_4_)_2_SO_4_, KNO_3_, or NaNO_2_. The pH of the media was adjusted with 6 mol/L HCl or 10 mol/L NaOH to 6.0, 7.0, 8.0, 9.0, and 10.0, respectively. The culture temperature was set at 15 °C, 20 °C, 28 °C, 37 °C, and 40 °C, and the shaking speed was set at 0 rpm, 50 rpm, 100 rpm, 150 rpm, and 200 rpm. The culture samples were taken to measure the OD_600_ and the concentrations of NH_4_^+^-N, NO_3_^-^-N, NO_2_^-^-N, TN, As(III), and As(V) at 20 h. All of the above experiments were performed in triplicate.

**Proteomics preparation and analysis**

The cells were collected by centrifugation (8000 g, 5 min) for 8 h and then freeze-dried. For total protein extraction, the cells were lysed in 200 μL of L3 buffer (7 M urea, 2 M thiourea, 4% SDS, 40 mM Tris-HCl pH 8.5, 1 mM phenylmethanesulfonyl fluoride, and 2 mM EDTA) for 5 min on ice, and 10 mM dithiothreitol (DTT) was added, followed by sonication for 15 min on ice and then centrifugation at 13000 g at 4 °C for 20 min. The supernatant was added to cold acetone overnight at -20 °C. The protein precipitates were collected by centrifugation and then resuspended with 8 M urea/100 mM triethylamine borane (TEAB) (pH 8.0) and 10 mM DTT at 56 °C for 30 min. After that, 55 mM iodoacetamide was added and placed in the dark at room temperature for 30 min to carry out an alkylation reaction. The protein concentration was determined by the Bradford method (Bradford, 1976). The protein (100 μg) was diluted fivefold with 100 mM TEAB, and then trypsin was added at a mass ratio of 1:50 (trypsin:protein) and incubated overnight at 37 °C. The peptides were desalted on a Strata-X C18 column and then freeze-dried in a vacuum.

The above peptides were resolved in 20 µL 0.5 M TEAB and then labeled by isobaric tags for relative and absolute quantitation (iTRAQ) according to the Reagent-8 plex Multiplex Kit (AB Sciex U.K. Limited). The mixed peptides were then fractionated by an Ultimate 3000 HPLC system (Thermo Dinoex, USA) using a Durashell C18 column (5 μm, 100 A, 4.6×250 mm). Twelve fractions were collected and desalted by a Strata-X column for further analysis. All peptide samples were analyzed using a Q Exactive Plus mass spectrometer (Thermo Fisher) coupled with the UltiMate 3000 RSLC nano system (Thermo Fisher). The MS/MS data were analyzed in Proteinpilot Software v4.5. The Paragon algorithm (Shilov et al., 2007) was used for protein identification by searching against the UniProt/SwissProt *Hydrogenophaga* databases. At least two peptides were required for the identification of the protein, which was further analyzed by GO, NCBI, COG, and KEGG data for their biological and functional properties. The significantly differentially expressed proteins were upregulated by ≥1.5 or downregulated by ≤−1.5 with a p-value of ≤0.05.

**Real-time quantitative PCR (qRT-PCR)**

Overnight cultured strains (OD_600_ = 1.0) were inoculated into 100 mL BM medium with or without the addition of 30 mg/L As(III) at 28 °C with shaking at 150 rpm. Samples were then collected at 4 h to extract the total RNA by TRIzol (Invitrogen) following incubation with RNase-free DNase I (Takara) at 37 °C to remove genomic DNA. The above samples were stopped by adding 50 mM EDTA at 65 °C for 10 min, and the concentration of RNA was measured by spectrophotometry (NanoDrop 2000, Thermo). Finally, 300 ng of total RNA was reverse transcribed into cDNA with a Revert Aid First Strand cDNA Synthesis Kit (Thermo) and then mixed with SYBR® Green Realtime PCR Master Mix (Toyobo). Quantitative RT-PCR was performed by ABI VIIA7 in a 0.1 mL Fast Optical 96-well Reaction Plate (ABI).


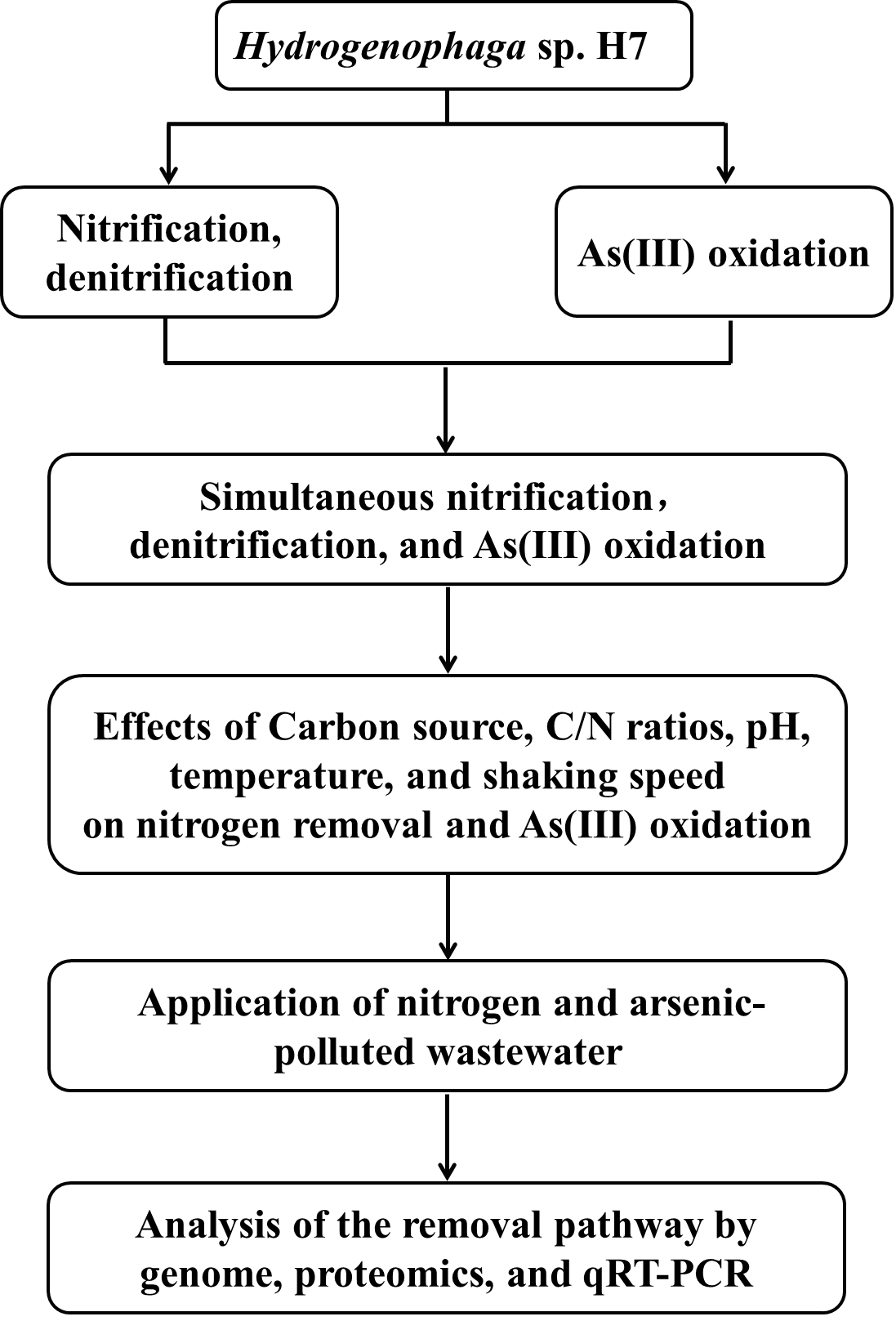


**Fig. S1 The experimental scheme of this study.**


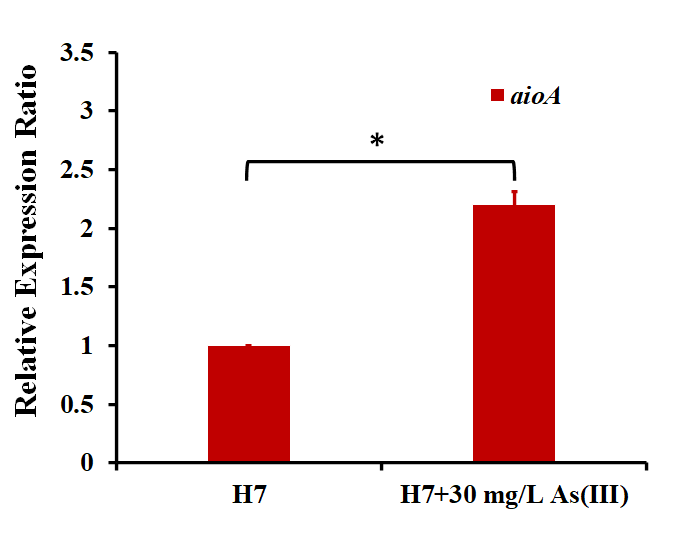


**Fig. S2 The relative expression of aioA by qRT-PCR.** * indicates the p value of ≤ 0.05. Error bars represent the mean ± standard deviation (n = 3).

| **Table S1 The primers used** **in Real-time quantitative PCR** | | | | |
| --- | --- | --- | --- | --- |
| | **Primers** | **Sequence** | **Fuction** | | --- | --- | --- | | 16S-RT-F/16S-RT-R | AGCGGTGGATGATGTGG  /GCTCGTTGCGGGACTTA | qRT-PCR for 16S rRNA gene | | aioA-RT-F/aioA-RT-R | GTTTGCCACCACCCTGACC  /GATGCCGTCGTGCGTGTA | qRT-PCR for aioA rRNA gene |   **Table S2 The removal efficiency and rate of N from NM by strain H7** | | | | |
|  | **0 h (mg/L)** | **24 h (mg/L)** | **Efficiency (%)** | **Maximum Rate (mg/L*h)** |
| NH_4_^+^-N | 21.73 ± 0.11 | 0.10 ± 0.05 | 99.50 | 2.71 |
| NO_3_^-^-N | 0.00 | 0.00 | 0.00 | 0.00 |
| NO_2_^-^-N | 0.00 | 0.00 | 0.00 | 0.00 |
| TN | 21.98 ± 0.35 | 0.59 ± 0.09 | 97.31 | 2.34 |

| **Table S3 The removal efficiency and rate of N from DM-1 by strain H7** | | | | |
| --- | --- | --- | --- | --- |
|  | **0 h (mg/L)** | **24 h (mg/L)** | **Efficiency (%)** | **Maximum Rate (mg/L*h)** |
| NH_4_^+^-N | 0.00 | 0.00 | 0.00 | 0.00 |
| NO_3_^-^-N | 15.00 ± 0.03 | 0.03 ± 0.01 | 99.82 | 1.53 |
| NO_2_^-^-N | 0.00 | 0.00 | 0.00 | 0.00 |
| TN | 15.75 ± 0.55 | 0.36 ± 0.14 | 97.71 | 2.16 |

| **Table S4 The removal efficiency and rate of N from DM-2 by strain H7** | | | | |
| --- | --- | --- | --- | --- |
|  | **0 h (mg/L)** | **24 h (mg/L)** | **Efficiency (%)** | **Maximum Rate (mg/L*h)** |
| NH_4_^+^-N | 0.00 | 0.00 | 0.00 | 0.00 |
| NO_3_^-^-N | 0.00 | 0.00 | 0.00 | 0.00 |
| NO_2_^-^-N | 21.43 ± 0.13 | 0.00 | 100.00 | 1.95 |
| TN | 22.50 ± 0.46 | 0.62 ± 0.29 | 97.26 | 1.92 |

| **Table S5 Comparison results of nitrogen removal efficiency** | | | |
| --- | --- | --- | --- |
|  | NH_4_^+^-N | NO_3_^-^-N | NO_2_^-^-N |
| *Hydrogenophaga* sp. H7 | 2.71 mg/L*h | 1.53 mg/L*h | 1.95 mg/L*h |
| *Pseudomonas* sp. ADN-42 | 0.43 mg/L*h |  |  |
| *Pannonibacter phragmitetus* B1 | 1.16 mg/L*h | 0.81 mg/L/h | 0.77 mg/L*h |
| *Pseudomonas tolaasii* Y-11 | 2.04 mg/L*h |  |  |
| *Pseudomonas putida* P1 |  | 0.68 mg/L*h |  |
| *Rhodococcus* sp. CPZ24 |  | 0.93 mg/L*h |  |
| *Pseudomonas* sp. yy7 |  |  | 0.76 mg/L*h |
| *Acinetobacter* sp. T1 |  |  | 1.69 mg/L*h |

| **Table S6 The removal efficiency and rate of N from SDM-1 by strain H7** | | | | |
| --- | --- | --- | --- | --- |
|  | **0 h (mg/L)** | **24 h (mg/L)** | **Efficiency (%)** | **Maximum Rate (mg/L*h)** |
| NH_4_^+^-N | 11.36 ± 0.02 | 0.04 ± 0.01 | 99.67 | 1.43 |
| NO_3_^-^-N | 7.50 ± 0.01 | 0.00 | 100.00 | 0.88 |
| NO_2_^-^-N | 0.00 | 0.00 | 0.00 | 0.00 |
| TN | 18.86 ± 0.26 | 0.34 ± 0.05 | 98.43 | 1.46 |

| **Table S7 The removal efficiency and rate of N from SDM-2 by strain H7** | | | | |
| --- | --- | --- | --- | --- |
|  | **0 h (mg/L)** | **24 h (mg/L)** | **Efficiency (%)** | **Maximum Rate (mg/L*h)** |
| NH_4_^+^-N | 11.36 ± 0.02 | 0.00 | 99.53 | 1.62 |
| NO_3_^-^-N | 0.00 | 0.00 | 0.00 | 0.00 |
| NO_2_^-^-N | 10.71 | 0.00 | 100.00 | 2.33 |
| TN | 22.08 ± 0.46 | 0.46 ± 0.06 | 97.90 | 1.60 |

| **Table S8 The removal efficiency and rate of N and As(III) from industrial wastewater by strain H7** | | | | |
| --- | --- | --- | --- | --- |
|  | **0 (mg/L)** | **16 h (mg/L)** | **Efficiency (%)** | **Maximum Rate (mg/L*h)** |
| NH_4_^+^-N | 20.21 ± 0.21 | 0.38 ± 0.03 | 98.11 | 1.91 |
| NO_3_^-^-N | 12.38 ± 0.33 | 0.23 ± 0.06 | 98.12 | 2.26 |
| TN | 35.60 ± 0.55 | 1.47 ± 0.30 | 95.87 | 3.06 |
| As(III) | 5.00 ± 0.01 | 0.00 | 100.00 | 0.86 |

**Table S9 Predicted proteins involved in nitrogen metabolism and arsenite oxidation**

| **Protein** | **Locus_tag of the predicted protein** |
| --- | --- |
| **Aissimilatory nitrate reduction** |  |
| Nitrate reductase (NasA) | BC358_05420 |
| Nitrite reductase (NirA) | BC358_07890 |
| Nitrite reductase (NirBD) | BC358_05410; BC358_05415 |
| **Denitrification** |  |
| Aerobic nitrate reductase (NapA) | BC358_05420 |
| Anaerobic nitrate reductase (NarGHI) | BC358_00350; BC358_00355; BC358_00365 |
| Nitrite reductase (NirS) | BC358_15830, BC358_15815, BC358_15790 |
| Nitric oxide reductase (NorBC) | BC358_15760 |
| Nitrous-oxide reductase (NosZ) | BC358_04445 |
| Arsenite oxidase large subunit (AioA) | BC358_19950 |
| Arsenite oxidase small subunit (AioB) | BC358_19945 |

| **No.** | **Accession no.** | **Name** | **Fold Change (*p*-value)** |
| --- | --- | --- | --- |
|  |  |  | **NH_4_^+^ vs Urea** |
|  | **Denitrification** |  |  |
| 2089 | BC358_05420 | NapA | **-1.9 (0.009)** |
| 2639 | BC358_00350 | NarG | **-2.0 (0.006)** |
| 2640 | BC358_00355 | NarH | **-1.5 (0.006)** |
| 2642 | BC358_00365 | NarI | **-1.7 (<0.001)** |
| 405 | BC358_15830 | NirS | **3.3 (0.003)** |
| 399 | BC358_15790 | NirS | **2.4 (0.004)** |
| 397 | BC358_15760 | NorB | **5.3 (0.003)** |
| 398 | BC358_15765 | NorC | **2.5 (0.002)** |
| 1957 | BC358_04445 | NosZ | **1.8 (0.009)** |

**Table S10. Proteins with different expression ratios based on** **iTRAQ proteomics**
